# Comparison of intraocular pressure andvault after implantation of implantable collamer lens with and without a central hole

**DOI:** 10.1186/s12886-016-0375-1

**Published:** 2016-11-16

**Authors:** Haiting Chen, Guangzeng Niu, Yuxiang Fan, Jingxue Ma

**Affiliations:** 1 Department of Ophthalmology, Cangzhou CentralHospital, Hebei, China; 2 Department of Ophthalmology, Second Hospital of Hebei Medical University, Hebei, China

**Keywords:** Intraocular Pressure, Vault, Implantable Collamer Lens

## Abstract

**Backgroud:**

The Implantable Collamer Lens (ICL) has been used widely for refractive errors, We performed this prospective randomized comparative study to compare postoperative intraocular pressure (IOP) and vaults of the eyes implanted with conventional ICL and central hole ICL.

**Methods:**

This study evaluated 44 eyes of 22 patients who underwent central hole ICL implantation in one eye and conventional ICL implantation in the other eye by randomization assignment. noncontact intraocular pressure were performed on 6 h, 1 day, 3 days, 1 week, 2 weeks, 1 month, 3 months and 6 months, while ICL vaults were measured on 1 day, 1 week, 1 month and 6 months.

**Results:**

The IOP of both eyeswithcentral hole and conventional ICLrosetemporarily during the first month after surgeries, especially on 1 day and 2 weeks points postoperatively. The IOP ofeyes with central hole ICL was higher than that of conventionl ICL. The vaults ofeyes with central hole and conventional ICL decreased slightly with time but did not significantly affect the postoperative IOP.

**Conclusions:**

Despite the sensitivity of viscoelastic agents or inflammation, this newly developed central hole ICL implantation appears to be equivalent in safty and effcacy to conventional ICL implantation for the correction of ametropia.

**Trial registration:**

Current Controlled Trials ChiCTR-INR-16008896. Retrospectively registered 24 July 2016.

**Electronic supplementary material:**

The online version of this article (doi:10.1186/s12886-016-0375-1) contains supplementary material, which is available to authorized users.

## Background

The Visian Implantable Collamer Lens (ICL) (STAAR Surgical Co.), a posterior chamber phakic intraocular lens (PIOL), has been reported to perform well for the correction of moderate to high ametropia [1–4]. However, in order to reduce the occurrence of pupillary block, this surgical technique requires preoperative laser iridotomy or intraoperative peripheral iridectomy, which is sometimes accompanied with iris hemorrhage, inflammation, or cataract [5]. Despite these additional procedures, some studies described cases of pupillary block, possibly because of the closure of previously iridotomies attributable to chronic inflammatory responses [6–8]. Moreover, the risk of cataract formation is presumed resulting from direct physical contact between the ICL and the crystalline lens or from malnutrition attributable to poor circulation of the aqueous humor [9].

Recently, the V4c Visian ICL (STAAR Surgical Company, Monrovia, California, USA) was designed with a 360 mm central hole to allow aqueous humor to flow which did not require additional peripheral iridotomies, and may reduce the risk for cataract formation [10]. Earlier studies have demonstrated that the V4c ICL has shown comparable refractive results to the conventional V4b ICL without central hole in vitro and in vivo experiments [11–13].

Considering that the presence of the central artificial hole induces a change in the aqueous humor dynamics [14], the central hole may affect the intraocular pressure (IOP) and the vault of ICL, which play a vital role in determining the safety of the ICL implantation technique. Although several studies have reported the IOP changes after implantation of v4c ICL, most of them described the situations in the middle or late stage postoperatively and are not in great detail [15, 16]. Moreover, the vault is a possible source of IOP changes, and it is uncertain whether there is any correlation between them in v4c implanted eyes. Therefore, in this prospective and random study, we examine and compare the IOP and vaults in the eyes with v4b and v4c ICL early postoperatively, then make further efforts to find out the relationship between IOP and vaults.

## Methods

This prospective randomized controlled trial was performed in Ophthalmological Institute of Cangzhou central hospital (Hebei, China) from January, 2016 to June, 2016. The study followed the tenets of the Declaration of Helsinki and was approved by an institutional review board. Written informed consent was obtained from all patients after they received a full explanation of the nature and possible consequences of the study.

Patients were selected following these inclusion criteria: a corrected distance visual acuity (CDVA) of 20/40 or better, stable bilateral myopia for at least 2 years, refractive error in the correctable range (from -0.50 to -18.00 diopters [D]), and a clear central cornea. The exclusion criteria included age younger than 20 years, anterior chamber depth (ACD) ≤3.0 mm, endothelial cell density (ECD) ≤ 2000 cell/mm2, mesopic pupil > 7.0 mm, cataract, history of glaucoma or other ophthalmic diseases.

Each patient underwent bilateral implantation of the posterior chamber phakic implantable Collamer lens with and without a 0.36-mm central artificial hole (central hole ICL V4c and conventional ICL V4b, STAAR Surgical) for the correction of moderate to high myopia by the same surgeon (Fan Yuxiang). The order of the two methods and the eye treated were randomized using a random number table at the inclusion visit. Using an envelope technique, the patients and examiners were masked to the types of ICLs implanted.

Forty four eligible eyes of 22 patients (9 men and 13 women) were involved in this study. This sample size offered 90% statistical power at the 5% level in order to detect a 2.0 mmHg difference in intraocular pressure between the 2 groups, when the standard deviation (SD) of the mean difference was 2.0 mmHg.

### Preoperative assessment

Before ICL implantation, patients underwent a series of ophthalmologic tests including uncorrected distance visual acuity (UDVA), corrected distance visual acuity (CDVA), manifest and cycloplegic refractions, slit-lamp examination, IOP measurement (noncontact tonometer, NCT Nidek Co, Ltd, Japan), Central corneal thickness (ultrasound pachymetry), ECD measurement, ultrasound biomicroscope (UBM), and dilated fundus examination. All eyes were targeted for emmetropia. ICL power calculation was performed according to the manufacturer’s instructions using a modified vertex formula. The size of the ICL was selected based on the horizontal white-to-white diameter measured by a metal caliper, and the anterior chamber depth was measured by scanning-slit topography (Pentacam; Oculus GmbH, Wetzlar, Germany).

### Surgical procedures

Peripheral iridotomies were performed at the 10:30 and 1:30 clock positions using a neodymium-doped yttrium-aluminum-garnet (Nd:YAG) laser for eyes scheduled to receive the V4b ICL, and eyes planned for V4c ICL implantation were exempt from this procedure. Both eyes of each patient underwent the same procedure. One fixed operator gave every eye pilocarpine drops, surface anesthesia, put on iridotomy contact len, then the laser was activated at a setting of 200 to 250mW on the V4b eye while 0mW on the V4c eye. The patients were uncertain which eye got laser iridotomy. Sixty minutes before surgery, pupils were dilated with dilating and cycloplegic agents. Under topical anesthesia, a model V4 ICL (central hole or conventional ICL) was inserted through a 3.0 mm clear corneal incision with the use of an injector cartridge (STAAR Surgical Co.) after injection of viscoelastics (Healon; Abbott Medical Optics, Santa Ana, California, USA) into the anterior chamber. The ICL was placed in the posterior chamber, and then viscoelastics was washed out of the anterior chamber with balanced salt solution, followed by instillation of miotic agent. After surgery, antibiotic (0.5% Levofloxacin Eye Drops, Santen Pharmaceutical Co.,Ltd) and steroidal (Tobramycin and Dexamethasone Eye Drops, Alcon NV) drugs were administered topically 4 times daily for 10 days and then gradually tapered.

### Postoperative assessment

Postoperative examinations of noncontact intraocular pressure were performed on 6 h, 1 day, 3 days, 1 week, 2 weeks, 1 month, 3 months and 6 months, while ICL vaults were measured on 1 day, 1 week, 1 month and 6 months. The evaluations of uncorrected distance visual acuity, best-corrected distance visual acuity and manifest refraction were also carried out. The examiners were only responsible for the examination of objective indicators without direct communications with patients.

### Statistical analysis

Data were analyzed using SPSS software (version 19.0, SPSS, Inc.). The results are expressed as mean ± standard deviation. The Wilcoxon signed rank test was used for statistical analysis to compare the differences between the 2 groups, and between preoperative and postoperative data in each group. One-way analysis of variance (ANOVA) was used to evaluate the changes over time. Meanwhile, the correlation between IOP and vaults was analyzed by Pearson correlation coefficient. A value of *P* < 0.05 was considered statistically significant.

## Results

The study sample comprised 44 eyes of 22 patients (9 men and 13 women). Table 1 shows the preoperative demographic characteristics and ICL parameters. Eyes had a baseline preoperative spherical equivalent (SE) of -9.64 ± 5.02 (-4.75,-15.75). 6 months after surgery, the SE was -0.50 ± 0.22 (-0.25,-1.00). At this time, 40(90.9%) eyes had an uncorrected distance visual acuity (UDVA) that was equal to or better than the preoperative corrected distance visual acuity(CDVA). One month after surgery while 44 (100%) eyes had a CDVA that was equal to or better than the preoperative CDVA. No eye lost 2 or more lines of CDVA. All surgical procedures were uneventful, and no postoperative complications such as cataract formation, pigment dispersion syndrome, papillary block, or axis rotation were seen during the 6-month observation period.

**Table 1:** Preoperative patient demographics in eyes undergoing implatation of V4b or V4c implantable collamer lenses

Demographic	V4b	V4c	P
Age (y)	26.5 ± 5.8(20,35)		
Sex (%female)	59.1		
Manifest spherical refraction (D)	-9.94 ± 4.88(-5.00,-15.50)	-9.43 ± 5.01(-4.75,-15.75)	0.564
Manifest cylinder (D)	-0.84 ± 1.05(0,-2.50)	-0.73 ± 0.71(0,-2.25)	0.673
UDVA (logMAR)	1.35 ± 0.23(1.00,2.00)	1.34 ± 0.22(1.00,2.00)	0.416
CDVA (logMAR)	-0.02 ± 0.04(-0.30,0.08)	-0.03 ± 0.05(-0.30,0.08)	0.807
White to white(mm)	11.8 ± 0.8(10.5-12.2)	11.7 ± 0.8(10.5-12.2)	0.476
Anterior chamber depth (mm)	3.43 ± 0.33(3.00-3.69)	3.42 ± 0.31(2.90-3.68)	0.761
ICL size (mm)	12.5 ± 0.8(11.0-13.0)	12.4 ± 0.8(11.0-13.0)	0.812

**Fig. 1:**
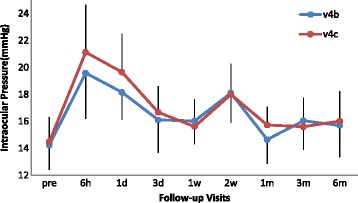
Time course of intraocular pressure changes in the perioperative period. The bars represent SD

The mean IOP of all eyes was 14.4 ± 1.8 mmHg (range 10.2–17.9 mmHg) before surgery. Postoperatively, the IOP level of two groups both shot up and reached the peak at 6 h, then decreased gradually until 1 week, following by a small increase on 2 weeks, declined to nearly preoperative levels on 1 month, at last maintained steadily during 1 to 6 months (Fig. 1). In conventional ICL (v4b) group, Postoperative IOP was higher than preoperative level except 1 month point. While in central hole ICL (v4c) group, Postoperative IOP was higher than preoperative level except 3 month point. As shown in Table 2, the mean IOP was 19.6 ± 3.4mmHg and 21.1 ± 3.5mmHg in the V4b and V4c group, respectively at 6 h postoperatively (*P* = 0.020). Meanwhile, on 1 day postoperatively, the mean IOP was 18.2 ± 2.0mmHg and 19.7 ± 2.9mmHg in the V4b and V4c group (*P* = 0.023). The IOP level in v4c group is a bit higher than that in v4b group, and these differences were statistically significant. However, there were no significant differences between the two groups at other follow-up visits (*P* > 0.05).

**Table 2:** Intraocular pressure (IOP) before and after the implantation of v4b and v4c ICL (X ± SD)

IOP(mmHg)	V4b	P_1_	V4c	P_2_	Z	P_3_
Pre	14.3 ± 1.8		14.5 ± 1.8		-1.271	0.204
6h	19.6 ± 3.4	0.000	21.1 ± 3.5	0.000	-2.329	0.020*
1d	18.2 ± 2.0	0.000	19.7 ± 2.9	0.000	-2.273	0.023*
3d	16.1 ± 2.4	0.003	16.7 ± 1.9	0.001	-0.958	0.338
1w	16.0 ± 1.7	0.003	15.6 ± 2.0	0.032	-0.779	0.436
2w	18.1 ± 2.2	0.000	18.0 ± 2.3	0.000	-0.417	0.676
1m	14.6 ± 1.7	0.399	15.7 ± 1.4	0.014	-1.901	0.057
3m	16.1 ± 2.2	0.001	15.6 ± 2.1	0.074	-0.520	0.603
6m	15.7 ± 2.4	0.009	16.0 ± 2.2	0.009	-0.422	0.673

P_1_: *p* value between preoperative intraocular pressure and postoperative intraocular pressure at different follow-up visits in V4b group

P_2_: *p* value between preoperative intraocular pressure and postoperative intraocular pressure at different follow-up visits in V4c group

P_3_: *p* value of intraocular pressure between V4b group and V4c group at different time points, *p <0.05

**Fig. 2:**
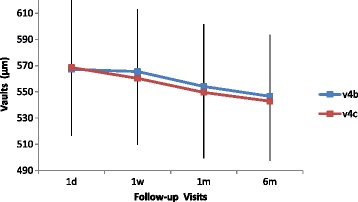
Time course of the vault changes after implantation of v4b and v4c ICL

**Fig. 3:**
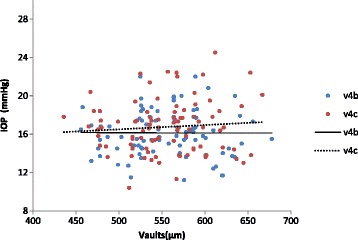
Correlation of the vault and intraocular pressrue after implantation of v4b and v4c ICL

The changes of the central hole and conventional ICL vaults with time are illustrated in Table 3. The differences between the two groups were not statistically significant at any time point after surgery. As shown in Fig. 2, the vaults in both groups displayed a mildly downward trend over time, but the variance was not statistically significant (*P* = 0.464 in v4b group, *P* = 0.330 in v4c group). Furthermore, there were no statistically significant correlations between the vaults and the IOP level in v4b group (Spearman correlation coefficient r = -0.007, *P* = 0.948) and v4c group (Spearman correlation coefficient r = 0.081, *P* = 0.454). (Fig. 3).

**Table 3:** Lens vaults after the implantation of v4b and v4c ICL (X ± SD)

Vaults(μm)	V4b	V4c	P
1d	567.2 ± 54.3	568.5 ± 51.9	0.626
1w	565.5 ± 47.7	560.2 ± 50.4	0.111
1m	554.1 ± 47.3	549.6 ± 50.0	0.121
6m	546.6 ± 46.7	542.8 ± 45.3	0.085

P: *p* value of the vaults between V4b group and V4c group at different time points

## Discussion

This trial was planned to find out the IOP changes and its correlation with vaults of central hole v4c ICL and conventional V4b ICL. A bilateral design, with similar myopia in both eyes of a given patient, comparing v4b ICL in one eye with v4c ICL in the other eye seemed to satisfy the criteria. Indeed, this preoperative, randomized study largely decreased the variability introduced by interindividual differences and differences in surgeons that linked to outcome measures. It also allowed a better statistical evaluation with each patient serving as his own control. Moreover, the patients and examiners were masked to the types of ICLs implanted, in order to reduce the information bias and subjective bias. Thus, for an identical number of cases, this methodology allowed us to define small quantitative differences more clearly. Furthermore, concerning the important problem of risks for the patient, because the long-term outcome of both ICL was unknown, our choice of performing a bilateral study balanced the potential risk for the patient.

In present study, we observed an increase IOP during the first month postoperatively with two transient peaks on the first 1 day and at approximately 2 weeks in both v4b and v4c groups. 11 eyes had an IOP over 22 mm Hg (4 in v4b group and 7 in v4c group) at 6 h and 4 eyes (all in v4c group) on 1 day point. The mean IOP of v4c group is significantly higher than that of v4b group at 6 h and 1 day postoperatively. However, there was no statistical difference between the vaults of the two groups on 1 day, and no chronically elevated IOP levels or papillary block were observed in any group. It demonstrated that the evaluated IOP was not caused by vault difference. In another study, Gonzalez-Lopez [17] found that obstruction of the trabecular meshwork or even of the central hole by residual viscoelastic material may be the main cause of this increase. Postoperative trabeculitis may also have affected early changes in IOP. Nevertheless, the reduction of surgical trauma and intraoperative time limited the degree of impact of the inflammation on IOP. In our study, the two groups had the similar ocular conditions and the same surgical equipmensts, which obviously reduced the interference of other factors. Therefore, we consider the fluid circulation function of central hole is less effective than that of peripheral iris incision, especially when the aqueous humor is mixed with the viscoelastic agent or a lot of inflammatory cells and pigments. After all, compared with the iris incision, the diameter of the central hole is smaller, and the number is only one.

During 3 days–6 months, the IOP in two groups showed an analogous trend and had no significant difference statistically. 1 eye had an IOP over 22 mmHg in each group on 2 weeks point requiring temporary topical hypotensive treatment. Consistent with other studies [18, 19], we attribute the increase in IOP during the first month after surgery to the effect of postoperative inflammation and topical steroids. As we use Tobramycin and Dexamethasone Eye Drops 4 times daily for 10 days, this steroid-related increases in IOP were most obviously 2 weeks after surgery correspondingly. Furthermore, with the decrease of the dosage of the drug and the intraocular inflammation, the IOP gradually went down to nearly preoperatively level. At the end of our study, only 1 eye had more than a 5 mmHg increase in IOP over the preoperative value (from 11.8 to 17.4 mmHg). However, this increases was not considered clinically relevant. After topical steroid treatment, no eye required further hypotensive treatment to maintain IOP. Thus, it can be seen that the effects of conventional drugs and mild inflammation are proximal on v4b and v4c ICL. Despite the absence of peripheral iridotomy, normal aqueous flow seems to occur as a result of the central hole. Hence, the new V4c ICL could maintain physiologic IOP levels under normal circumstances during the follow-up period.

The results of vaults in this study showed that in both central hole ICL and conventional ICL groups, vaults over the crystalline lens decreased slightly over time, although the variance was not statistically significant. And there were no significant between-group differences in the amount of vaults at any timepoint after surgery, suggesting that the time course of the central hole ICL vault is essentially equivalent to that of the conventional ICL. Kazutaka Kamiya’s findings were in line with our results in that there was a trend toward a decrease in the vault over time. Age-related increase in the thickness of the crystalline lens and the fixed position of the pIOL haptics may account for this slight decrease with time in the vault [20]. Hun Lee’s study also demonstrated that dynamic movement of the conventional and V4c ICL occur coincidentally with crystalline lens and anterior segment changes during the act of accommodation [21]. Therefore, the presence of an artificial hole does not significantly affect the amount of the vault. There are many factors affecting IOP. However, our results showed no significant correlation between the vault and the IOP, not only in eyes with v4b ICL but also in those with v4c ICL.

One main concern about the Visian V4c pIOL is the possibility of pupillary block caused by obstruction of the central hole which would affect IOP extremely. But no cases of pupillary block were observed in our study. The limitation of this study is that the sample data were limited. Only by increasing the number of implantions will we know whether the central hole ensures the free flow of aqueous humor in all possible clinical situations.

## Conclusion

Our comparative study demonstrated that the IOP of both central hole and conventional ICL evaluated temporary during the first month after surgeries, especially on 1 day and 2 weeks postoperatively. The IOP of central hole ICL was higher than that of conventionl ICL, indicating that the former is more vulnerable to viscoelastic agent or aqueous humor turbidity. In addition, the vaults of central hole and conventional pIOL decreased slightly with time and did not significantly affect the IOP postoperatively. Although longer observation in a large number of patients is required to assess the long-term qulity of this new ICL model, we agree with other authors [12, 16, 22] that this new surgical approach, which does not require additional iridectomies, is a safe and effective alternative to conventional ICL. Another advantage was that iridotomies were not needed because of the new ICL V4c design. Its drainage deficiencies may be made up possibly by meliorations.

## Additional files

Additional file 1: The IOP and valuts of each subject's eyes at different time points. (XLSX 18 kb)
Additional file 2: The mean value and standard deviation of IOP of each subject’s eyes at different time points. (XLSX 13 kb)

## Abbreviations

ACD: Anterior chamber depth
CDVA: Corrected distance visual acuity
ECD: Endothelial cell density
ICL: Implantable Collamer Lens
IOP: Intraocular pressure
PIOL: Posterior chamber phakic intraocular lens
SD: Standard deviation
UBM: Ultrasound biomicroscope
UDVA: Uncorrected distance visual acuity

## References

[1] Alfonso JF (2011). Posterior chamber collagen copolymer phakic intraocular lenses to correct myopia: five-year follow-up. J Cataract Refract Surg.

[2] Kamiya K (2009). Four-year follow-up of posterior chamber phakic intraocular lens implantation for moderate to high myopia. Arch Ophthalmol.

[3] Sanders DR (2004). United States Food and Drug Administration clinical trial of the Implantable Collamer Lens (ICL) for moderate to high myopia: three-year follow-up. Ophthalmology.

[4] Schallhorn S (2007). Randomized prospective comparison of visian toric implantable collamer lens and conventional photorefractive keratectomy for moderate to high myopic astigmatism. J Refract Surg.

[5] Siam GA (2008). Post-peripheral iridotomy inflammation in patients with dark pigmentation. Ophthalmic Surg Lasers Imaging.

[6] Smallman DS, Probst L, Rafuse PE (2004). Pupillary block glaucoma secondary to posterior chamber phakic intraocular lens implantation for high myopia. J Cataract Refract Surg.

[7] Park IK, Lee JM, Chun YS (2008). Recurrent occlusion of laser iridotomy sites after posterior chamber phakic IOL implantation. Korean J Ophthalmol.

[8] Chan KC (2008). Acute angle closure after implantable contact lens insertion unresponsive to surgical peripheral iridectomy. J Cataract Refract Surg.

[9] Chen LJ (2008). Metaanalysis of cataract development after phakic intraocular lens surgery. J Cataract Refract Surg.

[10] Fujisawa K (2007). Changes in the crystalline lens resulting from insertion of a phakic IOL (ICL) into the porcine eye. Graefes Arch Clin Exp Ophthalmol.

[11] Perez-Vives C (2013). Optical quality comparison of conventional and hole-visian implantable collamer lens at different degrees of decentering. Am J Ophthalmol.

[12] Alfonso JF (2013). Clinical outcomes after implantation of a posterior chamber collagen copolymer phakic intraocular lens with a central hole for myopic correction. J Cataract Refract Surg.

[13] Shimizu K (2012). Early clinical outcomes of implantation of posterior chamber phakic intraocular lens with a central hole (Hole ICL) for moderate to high myopia. Br J Ophthalmol.

[14] Kawamorita T, Uozato H, Shimizu K (2012). Fluid dynamics simulation of aqueous humour in a posterior-chamber phakic intraocular lens with a central perforation. Graefes Arch Clin Exp Ophthalmol.

[15] Higueras-Esteban A (2013). Intraocular pressure after implantation of the Visian Implantable Collamer Lens With CentraFLOW without iridotomy. Am J Ophthalmol.

[16] Shimizu K (2012). Intraindividual comparison of visual performance after posterior chamber phakic intraocular lens with and without a central hole implantation for moderate to high myopia. Am J Ophthalmol.

[17] Gonzalez-Lopez F (2013). Intraocular pressure during the early postoperative period after 100 consecutive implantations of posterior chamber phakic intraocular lenses with a central hole. J Cataract Refract Surg.

[18] Fernandes P (2011). Implantable collamer posterior chamber intraocular lenses: a review of potential complications. J Refract Surg.

[19] Jimenez-Alfaro I (2001). Safety of posterior chamber phakic intraocular lenses for the correction of high myopia: anterior segment changes after posterior chamber phakic intraocular lens implantation. Ophthalmology.

[20] Kamiya K (2015). Comparison of vault after implantation of posterior chamber phakic intraocular lens with and without a central hole. J Cataract Refract Surg.

[21] Lee H (2015). Effect of Accommodation on Vaulting and Movement of Posterior Chamber Phakic Lenses in Eyes With Implantable Collamer Lenses. Am J Ophthalmol.

[22] Huseynova T (2014). Comparative study of 2 types of implantable collamer lenses, 1 with and 1 without a central artificial hole. Am J Ophthalmol.

